# Measuring discrimination experienced by people with a mental illness: replication of the short-form DISCUS in six world regions

**DOI:** 10.1017/S0033291722000630

**Published:** 2023-07

**Authors:** Elaine Brohan, Graham Thornicroft, Nicolas Rüsch, Antonio Lasalvia, Megan M. Campbell, Özden Yalçınkaya-Alkar, Mariangela Lanfredi, Susana Ochoa, Alp Üçok, Catarina Tomás, Babatunde Fadipe, Julia Sebes, Andrea Fiorillo, Gaia Sampogna, Cristiane Silvestre Paula, Leonidas Valverde, Georg Schomerus, Pia Klemm, Uta Ouali, Stynke Castelein, Aneta Alexová, Nathalie Oexle, Patrícia Neves Guimarães, Bouwina Esther Sportel, Chih-Cheng Chang, Jie Li, Chilasagaram Shanthi, Blanca Reneses, Ioannis Bakolis, Sara Evans-Lacko

**Affiliations:** 1 Centre for Global Mental Health, Health Services and Population Research Department, Institute of Psychiatry, Psychology and Neuroscience, King's College London, London, UK; 2 Centre for Implementation Science, Health Services and Population Research Department, Institute of Psychiatry, Psychology and Neuroscience, King's College London, London, UK; 3 Department of Psychiatry II, Ulm University and BKH Günzburg, Günzburg, Germany; 4 Section of Psychiatry, Department of Neuroscience, Biomedicine and Movement Sciences, University of Verona, Verona, Italy; 5 Department of Psychiatry and Mental Health, Faculty of Health Sciences, University of Cape Town, Cape Town, South Africa; 6 Department of Psychology, Rhodes University, Makhanda, South Africa; 7 Department of Psychology, Ankara Yıldırım Beyazıt University, Ankara, Turkey; 8 Unit of Psychiatry, IRCCS Istituto Centro San Giovanni di Dio Fatebenefratelli, Brescia, Italy; 9 Parc Sanitari Sant Joan de Déu, CIBERSAM, Barcelona, Spain; 10 Department of Psychiatry, Istanbul Faculty of Medicine, Istanbul, Turkey; 11 Department of Nursing Sciences, School of Health Sciences of Polytechnic Institute of Leiria, Leiria, Portugal; 12 Center for Innovative Care and Health Technology (ciTechCare), Polytechnic Institute of Leiria, Leiria, Portugal; 13 Center for Health Technology and Services Research (Innovation & Development in Nursing), University of Porto, Porto, Portugal; 14 Department of Psychiatry, Lagos University Teaching Hospital, Lagos, Nigeria; 15 Psychosomatic and Psychotherapy-Rehabilitation Department, National Medical Rehabilitation Institute Szanatórium u. 19. 1121 Budapest, Budapest, Hungary; 16 Department of Psychiatry, University of Campania, L. Vanvitelli, Naples, Italy; 17 Developmental Disorder Program, Mackenzie Presbyterian University, São Paulo, Brazil; 18 Department of Psychiatry, University of Leipzig Medical Center, Leipzig, Germany; 19 Department of Psychiatry, Medical Faculty, Greifswald University, Greifswald, Germany; 20 Department Psychiatry A, Razi Hospital La Manouba, Tunisia; 21 Faculty of Medicine of Tunis, University Tunis El Manar, Tunisia; 22 Lentis Research, Lentis Psychiatric Institute, Groningen, The Netherlands; 23 Faculty of Behavioural and Social Sciences, University of Groningen, Groningen, The Netherlands; 24 Department of Public Mental Health, National Institute of Mental Health, Klecany, Czech Republic; 25 Faculty of Social Sciences, Charles University, Prague, Czech Republic; 26 Department of Mental and Public Health, Faculty of Medicine, State University of Montes Claros, Montes Claros, MG, Brazil; 27 Department of Psychotic Disorders, GGZ Drenthe Mental Health Institute, Assen, The Netherlands; 28 Department of Psychiatry, Chi Mei Medical Center, Tainan, Taiwan; 29 Department of Health Psychology, Chang Jung Christian University, Tainan, Taiwan; 30 The Affiliated Brain Hospital of Guangzhou Medical University, Guangzhou, China; 31 Department of Psychiatry, Government Medical College, Nizamabad, Telangana State, India; 32 Institute of Psychiatry and Mental Health, Institute of Biomedical Research (IdISSC), San Carlos University Hospital, Complutense University, Madrid, Spain; 33 Department of Biostatistics and Health Informatics, Institute of Psychiatry, Psychology and Neuroscience, King's College London, London, UK; 34 Personal Social Services Research Unit, London School of Economics and Political Science, London, UK

**Keywords:** Discrimination, DISCUS, Global mental health, Psychometrics, Stigma

## Abstract

**Background:**

The Discrimination and Stigma Scale (DISC) is a patient-reported outcome measure which assesses experiences of discrimination among persons with a mental illness globally.

**Methods:**

This study evaluated whether the psychometric properties of a short-form version, DISC-Ultra Short (DISCUS) (11-item), could be replicated in a sample of people with a wide range of mental disorders from 21 sites in 15 countries/territories, across six global regions. The frequency of experienced discrimination was reported. Scaling assumptions (confirmatory factor analysis, inter-item and item-total correlations), reliability (internal consistency) and validity (convergent validity, known groups method) were investigated in each region, and by diagnosis group.

**Results:**

1195 people participated. The most frequently reported experiences of discrimination were being shunned or avoided at work (48.7%) and discrimination in making or keeping friends (47.2%). Confirmatory factor analysis supported a unidimensional model across all six regions and five diagnosis groups. Convergent validity was confirmed in the total sample and within all regions [ Internalised Stigma of Mental Illness (ISMI-10): 0.28–0.67, stopping self: 0.54–0.72, stigma consciousness: −0.32–0.57], as was internal consistency reliability (*α* = 0.74–0.84). Known groups validity was established in the global sample with levels of experienced discrimination significantly higher for those experiencing higher depression [Patient Health Questionnaire (PHQ)-2: *p* < 0.001], lower mental wellbeing [Warwick-Edinburgh Well-being Scale (WEMWBS): *p* < 0.001], higher suicidal ideation [Beck Hopelessness Scale (BHS)-4: *p* < 0.001] and higher risk of suicidal behaviour [Suicidal Ideation Attributes Scale (SIDAS): *p* < 0.001].

**Conclusions:**

The DISCUS is a reliable and valid unidimensional measure of experienced discrimination for use in global settings with similar properties to the longer DISC. It offers a brief assessment of experienced discrimination for use in clinical and research settings.

## Introduction

Discrimination has been defined as the behavioural aspect of stigma; the enactment of problems of knowledge (ignorance or misinformation) and problems of attitudes (prejudice) (Thornicroft, Rose, Kassam, & Sartorius, 2007). Discrimination represents a pervasive global violation of the human rights of individuals who are experiencing disability due to mental illness (Drew et al., 2011).

It is well-evidenced that parity of esteem is not reflected in the funding of mental health services and access to both general medical and mental healthcare treatment for individuals with a mental illness (Angermeyer, Matschinger, Link, & Schomerus, 2014; Docherty & Thornicroft, 2015; Hilton, 2016). This has led to a global treatment gap in conditions such as major depressive disorder where only 1 in 5 people in high-income and 1 in 27 in low/lower-middle-income countries receive a minimally appropriate level of treatment (Thornicroft et al., 2017). In this regard, mental illness stigma and discrimination impact notably on population health (Hatzenbuehler, Phelan, & Link, 2013).

Structural and social capital barriers are also present in employment, education, housing, child custody and criminal justice settings (Brouwers et al., 2016; Jeffery et al., 2013; Webber et al., 2014). In personal relationships individuals also experience discrimination ranging from micro-aggressions such as invalidation of experience to physical, emotional, financial and sexual abuse and exploitation (Barber, Gronholm, Ahuja, Rüsch, & Thornicroft, 2020; Bhavsar, Dean, Hatch, MacCabe, & Hotopf, 2019; Drew et al., 2011). This is further reflected in the role of fear in characterising the experiences of mental health services users (Sweeney, Gillard, Wykes, & Rose, 2015).

To evidence the discrimination experienced by individuals with a mental illness globally, a robust measurement approach is required. The Discrimination and Stigma Scale (DISC) was developed as part of the International Study of Discrimination and Stigma Outcomes (INDIGO) (Thornicroft, Brohan, Rose, Sartorius, & Leese, 2009), a global research collaboration to evidence these experiences. The most recent version of this scale, DISC-12, is a 32-item structured interview designed to assess the experienced and anticipated discrimination in people with a mental illness. DISC has shown strong psychometric properties (Brohan et al., 2013) and has been widely used in over 50 countries with individuals with schizophrenia (Thornicroft et al., 2009), depression (Lasalvia et al., 2013) and bipolar disorder (Farrelly et al., 2014). In the study by Farrelly et al. (2014), similar levels of experienced discrimination were reported by those with depression, bipolar disorder or schizophrenia, with 87% experiencing discrimination in at least one area of life in the previous year. DISC-12 also collects qualitative examples of the experiences that participants are referring to when they provide a rating for their experience of discrimination. Further detail on the conceptual underpinnings of DISC, and similarities and differences between DISC and other measures of stigma and discrimination, are available elsewhere (Brohan et al., 2013; Brohan et al., in press; Brohan, Slade, Clement, & Thornicroft, 2010).

The ability to distil a longer scale into a short-form which retains the core aspects of the original, and demonstrates appropriate psychometric properties, is highly advantageous when the administration of the full measure is not feasible or necessary. This is particularly pertinent in low resource settings and a necessary step in ensuring that barriers to the global assessment of stigma and discrimination are reduced by shortening measures where possible (Bakolis et al., 2019; Brohan et al., in press). Beidas et al. (2015), suggest that the following properties are key in measures for use in low resource setting: freely and easily accessible; brief; have established psychometric properties; and high relevance. DISC-Ultra Short (DISCUS) aligns closely with these requirements by reducing participant burden, while remaining free to use, easily accessible and highly relevant. This paper provides evidence on the psychometric properties.

A full discussion of the psychometric properties of stigma and discrimination measures is presented elsewhere (Brohan et al., in press). A short-form version of the DISC, DISCUS, has recently been developed based on a secondary analysis of INDIGO network data with the use of novel meta-analytic factor analysis methods to perform item reduction and examine measurement invariance in a global sample. This resulted in an 11-item version which was further tested in a secondary analysis of a diverse London-based sample (Farrelly et al., 2014). Excellent agreement was observed between corresponding experienced discrimination scores on the 21-item experienced discrimination scale of DISC-12 and short-form DISCUS (Pearson's correlation *ρ* = 0.95) (Bakolis et al., 2019). DISCUS was also found to have appropriate internal consistency (*α* = 0.87) and convergent validity [Brief Psychiatric Rating Scale (BPRS) *r* = 0.35, Internalised Stigma of Mental Illness (ISMI), *r* = 0.35] (Bakolis et al., 2019). The anticipated discrimination aspects of DISC-12 have previously been developed into a stand-alone measure of anticipated discrimination and are not the focus of this current work (Gabbidon, Brohan, Clement, Henderson, & Thornicroft, 2013).

This paper seeks to evaluate whether the psychometric properties the DISCUS can be replicated in a sample of individuals with a diagnosis of depression, bipolar disorder, schizophrenia or an anxiety disorder in 15 countries/territories across six global regions. This is the first time that primary data have been collected using the short-form DISCUS and builds on the secondary analysis conducted by Bakolis et al.

The aims of this study are:

1. To evaluate the psychometric properties of the DISCUS in a primary data sample including: (i) scaling assumptions or the extent to which each DISCUS item contributes to a unidimensional scale producing a total experienced discrimination score; (ii) construct validity or the ability of DISCUS to yield consistent, reproducible estimates of the construct by assessing hypothesised relationships with similar constructs (convergent validity) and within distinct groups e.g. clinically different subgroups (known groups); (iii) reliability or the ability of DISCUS to yield consistent estimates of the construct under consideration across item responses (internal consistency).
2. To provide evidence on the appropriateness of using DISCUS to measure the discrimination experienced by individuals with mental illness in each of six global regions and five diagnosis categories.

## Methods

### Participants

Participants were recruited from 15 countries/territories: Brazil, China, Czech Republic, Germany, Hungary, India, Italy, Netherlands, Nigeria, Portugal, South Africa, Spain, Taiwan, Tunisia, Turkey. Sites were participating in the INDIGO anti-stigma programme and recruited participants locally through health services or service user organisations as has been done previously in the original DISC study (Lasalvia et al., 2013; Thornicroft et al., 2009). More details on sites are presented in online Supplementary Table S1. Each site recruited a minimum of 30 participants. Inclusion criteria specified that participants were at least 18 years of age, were using secondary mental health services or had a clinician-diagnosed mental illness, and had the capacity to consent. Individuals with comorbid mental illness were included, but the sample excluded those who had a primary diagnosis of substance abuse, personality disorder, dementia or other neurological condition without a major mental illness.

### Data collection

Data collection took place in the period 2018–2019. Translation and cross-cultural adaptation of study measures were completed in line with the principles adopted in previous INDIGO network and partnership studies (Lasalvia et al., 2013; Thornicroft et al., 2009). This included translation, back translation and concept checking (Knudsen et al., 2000; Thornicroft & Evans-Lacko, 2016). Fourteen different language versions of DISCUS were developed in this study and all translated versions are freely available from the Indigo website (http://www.indigo-group.org/stigma-scales/).

The study was approved by the appropriate ethical review board in each of the sites and by King's College London. Participants provided written informed consent. Sites could choose to administer DISCUS by interview or in a patient-completed written format. The majority were completed by interview with only two sites (Netherlands, China) reporting some use of the patient-completed option. Participants completed the DISCUS and a range of additional measures to establish known groups and convergent validity. All measures are listed below.

### Measures

#### Discrimination and Stigma Scale (DISCUS)

The DISCUS contains 11 items which assess aspects of experienced discrimination (Bakolis et al., 2019). It is a short-form version of DISC-12. Further detail on the development and psychometric validation of DISC-12 and the process of item-reduction and development of DISCUS is provided elsewhere (Bakolis et al., 2019; Brohan et al., 2013; Thornicroft et al., 2009). Each DISCUS item is rated on a 4-point Likert scale anchored between 0 = not at all and 3 = a lot. A non-applicable response option was also available.

#### Stopped contact

Three items, adapted from DISC-12, were included to assess the extent to which individuals were socially excluded in various aspects of their personal life including stopped contact by friends; partners (boyfriend/girlfriend/husband/wife) and family members (Thornicroft et al., 2009). Each item rated on a 4-point Likert scale anchored at 0 = not at all and 3 = a lot. Higher scores indicate a greater level of reduced contact.

#### Stigma stress

A 2-item short version of the original 8-item Stigma Stress Scale (Rüsch et al., 2009) assessed perceptions of stigma as a stressor, based on the perception of harm and coping resources. One item assessed perceived stigma-related harm, the other item perceived resources to cope with the threat of stigma. Each item was rated on a 7-point anchored numerical response scale (NRS): 1 = strongly disagree to 7 = strongly agree. Stigma stress was then calculated as the difference score (harm item minus coping resource item), with higher scores from −6 to +6 indicating higher stigma stress.

#### Stigma consciousness

Three items were included to assess stigma consciousness or the extent to which an individual considers that there is a public stigma towards people with a mental health problem and whether it impacts their interactions. The items were adapted from the Brief Stigma Consciousness Scale (Pinel, 1999). Each item was rated on a 4-point Likert scale anchored at 1 = strongly agree and 4 = strongly disagree. Higher scores indicate a higher level of stigma consciousness.

#### Patient Health Questionnaire (PHQ-2)

The PHQ-2 is a short-form measure of depression (Kroenke & Spitzer, 2010). It contains the first 2 items of PHQ-9, which constitute the two core DSM-IV items for major depressive disorder. Each item is rated on a 4-point Likert scale anchored at 0 = not at all and 3 = nearly every day. The total score ranges from 0 to 6. Higher scores indicate higher levels of depressive symptoms. A score of >3 has been used as a cut-point when screening for major depressive disorder (Löwe, Kroenke, & Gräfe, 2005).

#### Beck Hopelessness Scale (BHS-4)

The BHS-4 is a short-form measure of hopelessness derived from the 20-item item BHS (Aish & Wasserman, 2001; Beck, Weissman, Lester, & Trexler, 1974). Each item is rated on a 6-point Likert scale anchored at 1 = strongly agree and 6 = strongly disagree. Total scores range from 4 to 24. Higher scores indicate a higher level of hopelessness. A score of 11 has been used as a cut-point indicating a higher level of hopelessness (Yip & Cheung, 2006).

#### Suicidal Ideation Attributes Scale (SIDAS)

The SIDAS is a five-item measure of suicidal ideation (Van Spijker et al., 2014). Each item is rated on an 11-point NRS anchored at 0 = never to 10 = always. Total SIDAS scores range from 0 to 50. A score of 21 has been used as a cut-point to indicate a high risk of suicide behaviour (Van Spijker et al., 2014).

#### Internalised Stigma of Mental Illness (ISMI-10)

The ISMI-10 is a 10-item short-form measure of internalised stigma derived from the 29-item ISMI (Boyd, Otilingam, & DeForge, 2014). Each item is rated on a 4-point Likert scale anchored at 1 = strongly agree and 4 = strongly disagree. Total scores range from 0 to 4 with higher scores indicating higher internalised stigma. A two-category cut-point method for interpretation of scores has been proposed with a total score of 1.00–2.50 indicating lower internalised stigma and 2.51–4.00 indicating high internalised stigma for the 29-item ISMI (Ritsher, Otilingam, & Grajales, 2003).

#### Warwick-Edinburgh Well-being Scale (WEMWBS)

The WEMWBS is a 14-item scale which assesses mental wellbeing (Tennant et al., 2007). Each item is scored on a 5-point frequency response Likert scale anchored at 1 = none of the time and 5 = all of the time. Total scores range from 14 to 70 with higher scores indicating higher positive mental well-being. A score of ≤42 on WEMWBS can be used as a cut-point to indicate low mental wellbeing consistent with estimates of clinically relevant levels of depression in the UK population as assessed in the CES-D (Stewart-Brown, Samaraweera, Taggart, Kandala, & Stranges, 2015).

#### Socio-demographic and illness-related variables

Participants completed self-report questions on socio-demographic and clinical variables. Socio-demographic variables included: gender, age, education, housing situation and employment. Illness-reported variables included self-reported diagnosis, age at first diagnosis and disclosure of diagnosis.

### Psychometric evaluation of DISCUS

Descriptive statistics were calculated for all included measures. For DISCUS, scores were calculated at both the item and the scale level. The psychometric analysis of DISCUS focused on three aspects: (1) confirming the scaling assumptions of DISCUS; (2) evaluating scale reliability; (3) evaluating construct validity.

When >0 <2 item scores were missing (49/1195 cases) then a total score was still calculated. When >2 item scores were missing the mean score was not calculated (10/1195 cases). As a sensitivity analysis, scores were also calculated using median imputation for non-applicable and missing responses where the median response of all answered items for that individual item were used in place of non-applicable and missing responses.

Country-level data were grouped into six regions according to the United Nations statistics division geoscheme (http://unstats.un.org/unsd/methods/m49/m49regin.htm). Grouping decisions were guided by maximising the sample size available within each region.

Analysis was performed using SPSS version 26 and Stata version 16.

#### Discus scaling assumptions

DISCUS is assumed to be unidimensional based on previous research (Bakolis et al., 2019). Polychoric correlations were calculated between each item pair to ensure that all items are providing distinct information. Items were then summed and averaged to calculate a DISCUS total score. Corrected item-total correlations <0.30 were used to indicate the unacceptable fit of the items with the DISCUS total score (Terwee et al., 2007).

Multi-group confirmatory factor analysis was conducted to identify if the same construct is being measured across regions. To evaluate the overall model fit, the comparative fit index (CFI) and the root mean square error of approximation (RMSEA) were calculated. A CFI value of greater than 0.90 indicates an adequate fit to the data (Hu & Bentler, 1999). A value of RMSEA < 0.05 indicates close fit, values between 0.05 and 0.08 suggest adequate model fit, and values >0.10 suggest poor model fit (Hu & Bentler, 1999). The standardised root means squared residual (SRMR) was also calculated to examine how the model functions to reproduce the relationships from the input covariance matrix with an SRMR of <0.1considered acceptable (Kline, 2015). Postestimation modification indices were examined to identify pairs of variables where allowing correlation of the error terms may improve model fit e.g. modification index <3.84 (Acock, 2013). The selection of included correlated error terms was not purely data-driven, and the conceptual rationale for each inclusion was discussed by the authors. A final model was then run.

To examine whether the same construct is being measured across diagnostic groups, a second model was run which used the diagnosis as the grouping variable rather than region. The diagnosis was categorised as: Depression, Anxiety Disorder, Schizophrenia, Bipolar Disorder or Do not know diagnosis. Participants who reported other primary diagnoses were excluded from this analysis, though are included elsewhere in this paper. This model included the correlated error terms identified in the region model.

#### Reliability

The reliability of DISCUS was assessed by considering internal consistency using Cronbach's *α* with a criterion of *α* > 0.70 indicative of appropriate internal consistency for each subscale (Cronbach, 1951). *α* > 0.90 were also flagged, as this may indicate item redundancy.

### Construct validity

Construct validity provides evidence that scores on an instrument are related to other measures, or participant characteristics in a hypothesised manner. Two aspects of construct validity were assessed: (1) convergent validity; (2) known groups method [See (Brohan et al., in press)]. In convergent validity analyses, the following relationships were hypothesised [high correlation ⩾0.50 <0.80, moderate correlation ≥0.30 <0.50, and lower correlation <0.30 (Cohen, 1988)]:

1. Moderate/high positive correlation between mean DISCUS score and ISMI-10 self-stigma total score
2. Moderate/high positive correlation between mean DISCUS and stopping-self total score
3. Moderate positive correlation between mean DISCUS score and stigma stress difference score
4. Moderate positive correlation between mean DISCUS score and stigma consciousness total score

The known-groups method assessed differences in scores between participants who differed on identified clinical variables. The criterion was considered to be met when significantly different DISCUS scores (defined as *p* value <0.05) were obtained between the sample subgroups. The following groups were considered:

1. PHQ-2 (lower depression score <3 *v.* higher depression score ⩾3)
2. BHS-4 (lower hopelessness <11 *v.* higher hopelessness >11)
3. SIDAS (not high-risk suicide behaviour <21 *v.* high-risk suicide behaviour ⩾21)
4. WEMWBS (higher mental wellbeing ⩾42 *v.* lower mental wellbeing <41)

## Results

### Sample characteristics

1195 people participated in this study. Socio-demographic and clinical characteristics are reported in Table 1. These are available by region in online Supplementary Table S1. Table 1 also presents descriptive statistics for each study measure with a categorical interpretation of scores based on cut-points is further presented where available. Scores suggest that 46.4% of the sample had low mood indicative of major depressive disorder using PHQ-2. According to BHS-4, 54.4% had higher levels of hopelessness and 12.4% were at high risk of suicidal behaviour using SIDAS. 31.3% reported high internalised stigma. WEMWBS scores suggested that 40% had low mental wellbeing.

**Table 1.** Demographic and clinical characteristics for study sample (*n* = 1195)

Variable	*N* (%) or Mean (s.d.)			Variable	*N* (%) or Mean (s.d.)		
Sex	Male		601 (50.3%)	Told anyone diagnosis	Yes		1010 (84.5%)
	Female		593 (49.6%)		Yes, something else		77 (6.4%)
					No		106 (8.9%)
Age			45.12 (12.15)	Hospital admission for psychiatric care	Yes		865 (72.4%)
Years of education			11.52 (3.88)		No		325 (27.2%)
Employment status	Full-time paid work		185 (15.5%)	Age at first treatment			28.03 (11.55)
	Part-time paid work		120 (10.0%)	Country	Brazil (2 sites)		131 (11.0%)
	Self-employed		36 (3.0%)		China		43 (3.6%)
	Unpaid/voluntary		52 (4.4%)		Czech Republic		42 (3.5%)
	Unemployed		230 (19.2%)		Germany (2 sites)		208 (17.4%)
	Retired		158 (13.2%)		Hungary		30 (2.5)
	Homemaker		74 (6.2%)		India		30 (2.5%)
	Student		68 (5.7%)		Italy (3 sites)		149 (12.5%)
	Illness/sick leave		196 (16.4%)		Netherlands (3 sites)		107 (9.0%)
	Other		97 (8.1%)		Nigeria		31 (2.6%)
Know diagnosis	Yes		1016 (85.0%)		Portugal		30 (2.5%)
	No		176 (14.7%)		South Africa		71 (5.9%)
Diagnosis as reported by participants^a^	Schizophrenia		320 (27.1%)		Spain (2 sites)		92 (7.7%)
	Depression		313 (26.5%)		Taiwan		45 (3.8%)
	Anxiety disorder		162 (13.7%)		Tunisia		30 (2.5%)
	Bipolar disorder		152 (12.9%)		Turkey (2 sites)		156 (13.1%)
	Psychosis		56 (4.7%)				
	PTSD		29 (2.5%)	Region	Africa		132 (11.0%)
	Personality disorder		31 (2.6%)		Asia		118 (9.9%)
	Schizoaffective disorder		33 (2.8%)		Western Europe		315 (26.4%)
					Eastern Europe		228 (19.1%)
	Other		75 (6.4%)		Southern Europe		271 (22.7%)
	Do not know		175 (14.8%)		Latin America		131 (11.0%)
Mean DISCUS^b^	*n* = 1185	Range: 0–3	0.60 (0.58)	Stigma Stress difference	*n* = 1184	−6 to 6	−0.54 (3.06)
PHQ total^b^	*n* = 186	Range: 0–6	2.71 (2.07)	Mean Stigma conscious.	*n* = 1179	1 to 4	2.47 (0.73)
PHQ categorical	Lower depression <3		630 (52.7%)	Stopped contact total	*n* = 1167	0 to 9	1.85 (2.26)
	Higher depression score ≥3		556 (46.5%)	ISMI-10 total	*n* = 1198	1 to 4.0	2.27 (0.57)
BHS-4 total	1175	Range: 4–24	11.58 (5.23)	ISMI categorical	Low internalised stigma (1.00–2.50)		817 (68.4%)
BHS-4 categorical	Higher hopelessness >11		520 (43.5%)				
	Lower hopelessness <11		655 (54.8%)		High internalised stigma (2.51–4.00)		372 (31.1%)
SIDAS total	*n* = 1108	Range: 0–50	8.49 (11.17)	WEMWBS total	*n* = 1195	14 to 70	44.41 (12.08)
SIDAS categorical	Not high risk suicide behaviour <21		958 (80.2%)	WEMWBS categorical	Lower mental wellbeing (0–41)		499 (42%)
	High risk suicide behaviour ≥21		150 (12.6%)		Higher mental wellbeing ≥42		691 (8%)

a Note as an inclusion criterion all patients had a clinician-reported primary diagnosis of either schizophrenia, depression, bipolar disorder or anxiety disorder. The categories provided here represent the diagnoses reported by participants.

b Median and inter-quartile range presented rather than mean (s.d.) due to non-normality of scores.

The median DISCUS score was 0.45 (Interquartile range: 0.82) with a possible range of 0–3. This ranged from 0.38 (0.41) in Southern Europe to 0.88 (0.69) in Latin America. The most frequently reported experiences of discrimination were being shunned or avoided at work by (49.6%) and discrimination in making or keeping friends (47.3%). 13 participants (1.09%) reported no experiences of discrimination across any all items. The frequency and percentage of response category endorsement for each DISCUS item by region is displayed in online Supplementary Table S2.

### Psychometric evaluation of DISCUS

The results of the psychometric evaluation of DISCUS are presented in Table 2. No differences in the interpretation of results were seen in the sensitivity analysis using median imputation to assign scores for non-applicable and missing responses.

**Table 2.** Summary of psychometric properties of DISCUS by region

Psychometric property	Constituent parts			Africa (*n* = 132)	Asia (*n* = 118)	Western Europe (*n* = 315)	E. Europe (*n* = 228)	S. Europe (*n* = 271)	Latin America (*n* = 131)	Total (*n* = 1195)
Scaling assumptions^a^	Inter-item polychoric correlations (Mean, range)			Mean = 0.47, −0.01–0.76	Mean = 0.46, 0.18–0.81	Mean = 0.48, 0.28–0.70	Mean = 0.33, 0.03–0.71	Mean = 0.33, −0.04–0.58	Mean = 0.44, 0.12–0.69	Mean = 0.43, 0.28–0.59
	Corrected Item-total correlations			0.29 to 0.62	0.37 to 0.67	0.38 to 0.59	0.25 to 0.61	0.16 to 0.58	0.38 to 0.65	0.38 to0.56
Reliability	Internal consistency			*α* = 0.84	*α* = 0.83	*α* = 0.82	*α* = 0.77^b^	*α* = 0.74	*α* = 0.83	*α* = 0.82
Construct Validity	Convergent validity^c^	DISCUS and ISMI-10		0.58**^d^	0.67**	0.41**	0.28**	0.29**	0.51**	0.41**
		DISCUS and stopping-self total		0.59**	0.66**	0.61**	0.67**	0.54**	0.72**	0.64**
		DISCUS and stigma stress difference		0.07NS	0.32**	0.22**	0.19**	0.42**	0.44**	0.29**
		DISCUS and stigma consciousness		0.45**	0.36**	0.32**	0.33**	0.33**	0.57**	0.38**
	Known groups method^e^ (Wilcoxon rank sum)	PHQ-2	Lower depression <3	0.18*^f^	0.36**	0.36**	0.45NS	0.27NS	0.36*	0.36**
			Higher depression ⩾3	0.55	0.73	0.73	0.55	0.27	0.82	0.64
		BHS-4	Lower hopelessness <11	0.23NS	0.55*	0.55NS	0.45*	0.18**	0.68NS	0.36**
			Higher hopelessness >11	0.32	0.45	0.64	0.64	0.36	0.82	0.55
		SIDAS	Not high risk suicidal behaviour <21	0.27NS	0.45NS	0.55**	0.45*	0.27NS	0.64**	0.45**
			High risk suicidal behaviour SIDAS ⩾21	0.27	0.91	0.90	0.72	0.41	1.41	0.82
		WEMWBS	Higher mental wellbeing ⩾42	0.18NS	0.45*	0.55*	0.45NS	0.27NS	0.59*	0.36**
			Lower mental wellbeing 0–41	0.36	0.73	0.73	0.55	0.27	0.82	0.64

a See Fig. 2 for results of multi-group confirmatory factor analysis.

b *α* increased to 0.78 with the removal of DISCUS item 4: housing. No other increases with item deletion were seen in any region.

c Spearman's *ρ* was used for convergent validity analysis due to non-normality of DISCUS mean scores.

d **Indicates *p* < 0.001, *indicates *p* < 0.05, NS indicates non-significant.

e Wilcoxon rank-sum test was used to compare median scores for known groups analysis due to non-normality of DISCUS mean scores.

f Median DISCUS scores are displayed with the significance level indicated on the upper result for each pair.

#### DISCUS scaling assumptions

Polychoric correlations did not indicate item-redundancy with an average correlation of 0.41 in the total sample (average range 0.33–0.47 across regions). The corrected item-total correlations were generally in the moderate range (0.36–0.57 in the total sample).

**Fig. 1.**
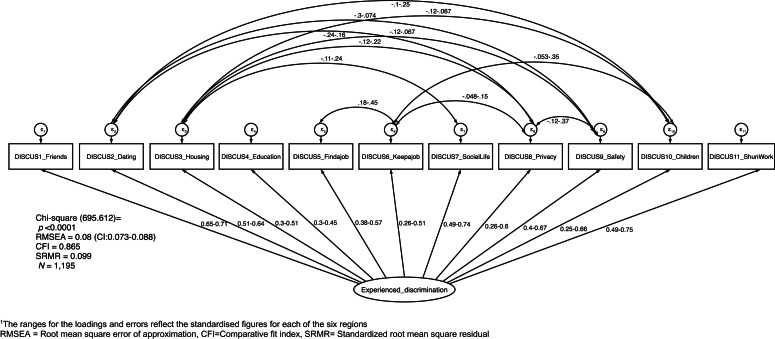
Multi-group CFA model for DISCUS by region^1^. ^1^The ranges for the loadings and errors reflect the standardised figures for each of the six regions. RMSEA, Root mean square error of approximation; CFI, Comparative fit index; SRMR, Standardised root mean square residual.

The 11 DISCUS items all loaded significantly on a single dimension with standardised loadings by region ranging from 0.29 to 0.49 for Item 4: Education to 0.49–0.74 for Item 7: Social life and 0.49 to 0.75 for Item 11: Shunned at Work. Significant loadings were seen for each item across each of the six regions. The one factor CFA solution for DISCUS had a reasonable fit, as represented in Fig. 1. The RMSEA value of 0.08, and SMSR <0.10 suggest acceptable model fit while the CFI is slightly lower than the threshold of 0.90, at 0.87. The significant χ^2^ test indicates the model is not a perfect fit. This model includes correlation of the following error terms (Item 2: Dating and Items 8: Privacy, 9: Safety and 10: Children), (Item 3: Housing and Items 7: Social life, 8: Privacy, 9: Safety and 10: Children), (Item 5: Finding work and Item 6: Keeping work), (Item 6: Keeping work and Item 8: Privacy, 10: Children), (Item 8: Privacy and Item 9: Safety). SMSR was also examined at the regional level with all regions having scores <0.1 (range 0.053–0.095) apart from Asia, SMSR = 0.157.

**Fig. 2.**
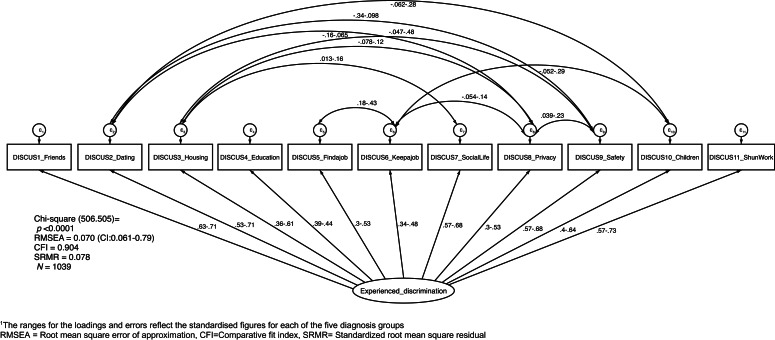
Multi-group CFA model for DISCUS by region and diagnosis category^1^. ^1^The ranges for the loadings and errors reflect the standardised figures for each of the five diagnosis groups. RMSEA, Root mean square error of approximation; CFI, Comparative fit index; SRMR, Standardised root mean square residual.

When this model was run with diagnosis as a grouping variable, rather than region, significant loadings were seen for each item across each of five diagnostic categories (Depression; Anxiety Disorder; Bipolar Disorder; Schizophrenia; do not know mental diagnosis). The one factor CFA solution for DISCUS is graphically represented in Fig. 2. The RMSEA of 0.070, CFI of 0.904 and SMSR <0.10 suggest acceptable model fit. SMSR was also examined by diagnosis category with all categories having scores <0.1 (range 0.060 to.084).

### Reliability

Cronbach's *α* coefficient for the eleven-item DISCUS was strong at 0.82. This ranged from 0.74 in Southern Europe to 0.84 in Africa. Alphas did not substantially increase with item deletion across any region.

### Construct validity

There was evidence of convergent validity in the total sample and in most regions. Moderate/high correlation was seen between mean DISCUS and stopping-self total score and moderate correlation between mean DISCUS score and stigma consciousness total score was achieved in the total sample and all regions fully meeting the threshold. Moderate correlations between mean DISCUS score and stigma stress/coping total score were achieved in the total sample but not all regions with scores lower in Western and Eastern Europe and non-significant in Africa. Moderate/high correlations were seen between mean DISCUS score and ISMI-10 self-stigma score in the total sample and all regions apart from Eastern Europe where scores were slightly lower at *ρ* = 0.28 and Eastern Europe *ρ* = 0.29.

The Wilcoxon rank-sum test was used to compare median scores for known groups analysis. There was evidence of construct validity using the known groups method in the total sample with a significant difference between groups seen for each hypothesised relationship. In each region, a minimum of 50% of the hypothesised group differences were observed.

## Discussion

DISC has been widely used to assess mental illness related experienced discrimination worldwide and provides a comprehensive picture of these experiences (Farrelly et al., 2014; Lasalvia et al., 2013; Thornicroft et al., 2009). Previous research suggests that DISCUS, an 11-item, short-form version developed and confirmed using secondary data, produces an experienced discrimination score which is equivalent to DISC-12 scores (Bakolis et al., 2019). This current study advances this work by providing evidence that the unidimensional nature of experienced discrimination, as assessed using DISCUS, is replicated in a primary data sample from 21 sites in 15 countries/territories encompassing six global regions. Unidimensionality was also supported across five diagnosis categories (Depression, Anxiety Disorder, Schizophrenia, Bipolar Disorder and ‘Do not know diagnosis)’. Further support for convergent validity was provided in relation to self-stigma, stopping oneself from engaging in social contact and stigma stress, across all six global regions. Internal consistency was strong across all regions. The known groups method also provided evidence that experienced discrimination is greater among those experiencing higher levels of depression (PHQ-2: *p* < 0.001), higher hopelessness (BHS-4, *p* < 0.001), higher risk of suicidal behaviour (SIDAS, *p* < 0.001) and lower mental wellbeing (WEMWBS, *p* < 0.001). This work is further supported by a sensitivity analysis using median imputation of DISCUS item scores. This resulted in a very limited change to the interpretation of results suggesting that the non-applicable and missing responses were not providing meaningfully different information whether they were scored as 0 ‘not at all’ or using the median value.

This suggests that DISCUS provides a short-form, reliable and valid approach for measuring experienced discrimination which performs similarly across a range of global regions and diagnosis groups. It is suitable for use where full information on the content of discrimination experiences (as provided by DISC) is not required or not feasible within the setting. It offers detail on the content of experiences within the most key items, as well as a mean item score which is highly comparable with that produced by the full version.

DISCUS draws on macro, meso and micro-level experiences of discrimination with a focus on micro-level experiences. The most frequently reported experiences in this study were ‘being shunned or avoided at work’ by 48.7% of global participants (Item 11) and ‘discrimination in making or keeping friends’ by 47.2% (Item 1). This is in keeping with previous studies (Bakolis et al., 2019). There is some variation across regions with ‘discrimination in keeping a job’ most frequently reported in Latin America. This provides support for interventions that focus on addressing loneliness [e.g. (Castelein, Bruggeman, Davidson, & Gaag, 2015; Vogel et al., 2019)] or employment challenges [e.g. (Janssens, van Weeghel, Henderson, Joosen, & Brouwers, 2020)] among individuals with a mental illness.

It is important to note that although a unidimensional model of experienced discrimination is appropriate across all regions, this does not mean that the nature of experiences is the same. This is supported by the region-level fit statistics which suggest that the model may fit less well for Asia with greater residual variance here, as indicated by the higher SRMR. A different pattern of non-applicable responses was noted in Asia which may contribute to this. For example, for the Education-related item (Item 4), 63.6% of responses were non-applicable with the next highest 46.9% for Southern Europe. Further examination of the open-text responses collected using DISCUS could help to explain some of the reasons for this difference but is beyond the scope of this current paper. Discrimination experiences are compounded in an intersectional way for individuals who experience discrimination because of other minority characteristics as well as mental illness including race/ethnicity, sexuality, gender, age, and comorbid mental or physical disabilities (Hall et al., 2014). Further work is still required to understand how these multiple discrimination experiences influence the safety, health and wellbeing of individuals with mental illness.

The correlated error terms most commonly included in the CFAs relate to Item 8: Privacy (correlated with Item 2: Dating, Item 3: Housing, Item 6: Keeping a job and Item 9: Safety) and Item 9: Safety (correlated with Item 2: Dating, Item 3: Housing, Item 8: Privacy). This fits with evidence that fear is a pervasive theme in understanding mental health service users experiences and suggests that privacy and safety violations may underlie experienced discrimination in other areas (Sweeney et al., 2015). Further analysis of the qualitative responses to understand the underpinning nature of safety and privacy concerns on other experiences of discrimination is an area for future research.

### Limitations

As diagnoses were self-reported by participants recruited through clinical settings, the extent to which diagnoses correspond to those recorded in clinical notes is unknown. However, as the focus of this work is to assess discrimination experiences then arguably it is most important to understand participants own understanding of their diagnosis as this is the information that they use in making sense of their experiences of discrimination.

Analysis using the known groups method did not reach significance across all regions. The unequal distribution of responses across categories within certain regions is likely to be a limiting factor. This is particularly true for SIDAS where low numbers selected a response indicating ‘high risk' e.g. *n* = 4 in Africa. As this is the most severe indicator of mental ill-health in the study then an unequal split in subgroup membership is to be expected, however, this is magnified at the regional level. Although significance was still found in 3 of the 6 regions, with scores in the expected direction for a further two regions, it may be most accurate to consider the total sample for these analyses.

There are also limitations in the use of a convenience sample of participants and in the regional groupings. Although established regional groupings have been used, these are broad categorisations to allow for an adequate sample size in each region (range 118–315). The validity of grouping diverse countries such as India, Taiwan and China under ‘Asia’ or Tunisia, Nigeria and South Africa under ‘Africa’ is uncertain; however, it reflects the regions currently used by the WHO. Moreover, confirmation of robust psychometrics across such broad groups is a strength.

Further work is also required to establish the ability to detect a change, and interpretation of scoring including minimal important change, for DISCUS if it is to be used as an outcome measure in interventional settings. However, information on these aspects is available for the full DISC.

## Conclusions

The 11-item DISCUS is a reliable, unidimensional, measure of experienced discrimination for use in global settings among individuals with a range of mental disorders. It is highly relevant with over 98% of participants reporting at least one experience of discrimination. It has confirmed construct validity in these groups using convergent validity and known groups methods, replicating earlier findings. It offers a brief, free and easily accessible, person-reported, measure for use in estimating the discrimination experienced by individuals with a mental illness. It is recommended as an evaluation tool in global settings to assess the impact of discrimination. DISCUS offers a scalable solution for discrimination measurement in busy, real-world, and resource-limited settings.

## Supplementary material

For supplementary material accompanying this paper visit https://doi.org/10.1017/S0033291722000630. click here to view supplementary material
