# Supplementary material for: Measuring discrimination experienced by people with a mental illness: replication of the short-form DISCUS in six world regions
Source: Psychol Med. 2022 Mar 30;53(9):3963–73. doi: 10.1017/S0033291722000630 (PMC10317801; doi:10.1017/S0033291722000630)
Supplement: Supplementary file 1 [file S0033291722000630sup001.docx]

**Supplementary Table 1**: Demographic and clinical characteristics for study sample by region

| **Variable** | **N(%) or Mean(SD)** | | | **Variable** | **N(%) or Mean(SD)** | | |
| --- | --- | --- | --- | --- | --- | --- | --- |
| **Region = Africa (n=132)** | | | | | | | |
| Sex | Male | | 101(76.5%) | Told anyone diagnosis | Yes | | 84(63.6%) |
|  | Female | | 31(23.5%) |  | Yes, something else | | 16(12.1%) |
| Age | | | 39.08(10.68) |  | No | | 32(24.2%) |
| Years of education | | | 10.73(4.1) | Hospital admission for psychiatric care | Yes | | 112(86.2) |
| Employment status | Full-time paid work | | 12(9.2%) |  | No | | 18(13.8%) |
|  | Part-time paid work | | 9(6.9%) | Age at first treatment | | | 24.69(7.81) |
|  | Self-employed | | 8(6.1.%) | Know diagnosis | Yes | | 95(72%) |
|  | Unpaid‎/ voluntary | | 0 |  | No | | 37(28.9%) |
|  | Unemployed | | 21(16%) | Diagnosis as reported by participants**^1^** | Schizophrenia | | 32(25%) |
|  | Retired | | 1(0.8%) |  | Depression | | 10(7.8%) |
|  | Homemaker | | 12(9.2%) |  | Anxiety disorder | | 0(0%) |
|  | Student | | 19(14.5%) |  | Bipolar disorder | | 22(17.2%) |
|  | Illness‎/sick leave | | 47(35.9.%) |  | Psychosis | | 2(1.6%) |
|  | Other | | 2(1.5%) |  | PTSD | | 0(0.%) |
| Country | Nigeria | | 31(23.5%) |  | Personality disorder | | 0(0%) |
|  | South Africa | | 71(53.8%) |  | Schizoaffective disorder | | 0(0%) |
|  | Tunisia | | 30(22.7%) |  | Other | | 25(25%) |
|  |  |  |  |  | Do not know | | 37(28.9%) |
| Mean DISCUS**^2^** | n=126 | Range: 0-2.82 | 0.46(0.55) | Stigma stress difference | n=129 | Range: -6-6 | -1.01(3.08) |
| PHQ total | n=132 | Range: 0-6 | 1.36(1.71) | Stigma conscious. total | n=129 | Range: 1-4 | 2.40(0.79) |
| PHQ categorical | Depression score <3 | | 103(78%) | Stopped self total | n=132 | Range: 0-9 | 1.38(2.15) |
|  | Depression score ≥3 | | 29(22%) | ISMI-10 total | n=128 | Range: 1-3.7 | 2.15(0.64) |
| BHS-4 total | n=130 | Range: 4-24 | 9.50(5.37) | ISMI categorical | Low internalized stigma 1.00-2.50 | | 95(74.2%) |
| BHS-4 categorical | Lower hopelessness <11 | | 80(61.5%) |  |  |  |  |
|  | Higher hopelessness >11 | | 50(38.5%) |  | High internalized stigma >2.50 | | 33(25.8%) |
| SIDAS total | n=126 | Range: 0-47 | 5.92(6.83) | WEMWBS total | n=121 | Range: 19-70 | 49.37(11.19) |
| SIDAS categorical | Not high risk suicide behaviour <21 | | 122(96.8%) | WEMWBS categorical | Lower mental wellbeing <41 | | 32(26.4%) |
|  | High risk suicide behaviour ≥21 | | 4(3.2%) |  | : Higher mental wellbeing ≥42 | | 89(73.6%) |

| **Variable** | **N(%) or Mean(SD)** | | | **Variable** | **N(%) or Mean(SD)** | | |
| --- | --- | --- | --- | --- | --- | --- | --- |
| **Region = Asia (n=118)** | | | | | | | |
| Sex | Male | | 56(47.9%) | Told anyone diagnosis | Yes | | 99(83.9%) |
|  | Female | | 61(52.1%) |  | Yes, something else | | 6(5.1%) |
| Age | | | 44.58(12.55) |  | No | | 13(11.0) |
| Years of education | | | 11.08(3.41) | Hospital admission for psychiatric care | Yes | | 88(74.6%) |
| Employment status | Full-time paid work | | 14(11.9%) |  | No | | 30(25.4%) |
|  | Part-time paid work | | 25(22.1%) | Age at first treatment | | | 26.28(8.85) |
|  | Self-employed | | 6(5.1%) | Know diagnosis | Yes | | 108(91.5%) |
|  | Unpaid‎/ voluntary | | 1(0.8%) |  | No | | 10(8.5%) |
|  | Unemployed | | 29(24.6%) | Diagnosis as reported by participants**^1^** | Schizophrenia | | 71(60.2%) |
|  | Retired | | 6(5.1%) |  | Depression | | 13(11%) |
|  | Homemaker | | 18(15.3%) |  | Anxiety disorder | | 12(10.2%) |
|  | Student | | 7(5.9%) |  | Bipolar disorder | | 10(8.5%) |
|  | Illness‎/sick leave | | 12(10.2%) |  | Psychosis | | 3(2.5%) |
|  | Other | | 0(0%) |  | PTSD | | 0(0%) |
| Country | Guangzhou- China | | 43(36.4%) |  | Personality disorder | | 0(0%) |
|  | Taiwan | | 45(38.1%) |  | Schizoaffective disorder | | 0(0%) |
|  | India | | 30(25.4%) |  | Other | | 2(1.7%) |
|  |  |  |  |  | Do not know | | 10(8.5%) |
| Mean DISCUS**^2^** | n=116 | Range: 0-3 | 0.52(0.57) | Stigma stress difference | n=118 | Range: -6-6 | -0.84(2.70) |
| PHQ total | n=117 | Range: 0-6 | 2.21(1.99) | Stigma conscious. total | n=118 | Range: 1-4 | 2.39(0.66) |
| PHQ categorical | Depression score <3 | | 72(61.5%) | Stopped self total | n=118 | Range: 0-9 | 1.34(1.92) |
|  | Depression score ≥3 | | 45(38.5%) | ISMI-10 total | n=118 | Range: 1-3.8 | 2.48(0.49) |
| BHS-4 total | n=116 | Range: 4-24 | 12.18(4.19) | ISMI categorical | Low internalized stigma 1.00-2.50 | | 70(59.3) |
| BHS-4 categorical | Lower hopelessness <11 | | 46(39.7%) |  |  |  |  |
|  | Higher hopelessness >11 | | 70(60.3%) |  | High internalized stigma >2.50 | | 48(40.7%) |
| SIDAS total | n=76 | 0-50 | 9.8(11.61) | WEMWBS total | n=118 | Range: 18-70 | 44.4(11.53) |
| SIDAS categorical | Not high risk suicide behaviour<21 | | 65(85.5%) | WEMWBS categorical | Lower mental wellbeing <41 | | 47(39.8%) |
|  | High risk suicide behaviour ≥21 | | 11(14.5%) |  | Higher mental wellbeing ≥42 | | 71(60.2%) |

| **Variable** | **N(%) or Mean(SD)** | | | **Variable** | **N(%) or Mean(SD)** | | |
| --- | --- | --- | --- | --- | --- | --- | --- |
| **Region = Western Europe (n=315)** | | | | | | | |
| Sex | Male | | 143(45.4%) | Told anyone diagnosis | Yes | | 284(90.2%) |
|  | Female | | 172(54.6%) |  | Yes, something else | | 21(6.7%) |
| Age | | | 47.14(12.75) |  | No | | 10(3.2) |
| Years of education | | | 12.36(3.49) | Hospital admission for psychiatric care | Yes | | 241(76.5) |
| Employment status | Full-time paid work | | 32(10.2%) |  | No | | 71(22.5%) |
|  | Part-time paid work | | 27(8.6%) | Age at first treatment | | | 29.67(12.97) |
|  | Self-employed | | 3(1.0%) | Know diagnosis | Yes | | 274(87.0%) |
|  | Unpaid‎/ voluntary | | 38(12.1%) |  | No | | 39(12.4%) |
|  | Unemployed | | 50(15.9%) | Diagnosis as reported by participants**^1^** | Schizophrenia | | 36(11.4%) |
|  | Retired | | 50(15.9%) |  | Depression | | 125(39.7%) |
|  | Homemaker | | 14(4.4%) |  | Anxiety disorder | | 44(14.0%) |
|  | Student | | 12(3.8%) |  | Bipolar disorder | | 27(8.6%) |
|  | Illness‎/sick leave | | 62(19.7%) |  | Psychosis | | 20(6.3%) |
|  | Other | | 38(12.1%) |  | PTSD | | 29(9.2%) |
| Country | Germany-Ulm | | 102(32.4%) |  | Personality disorder | | 21(6.7%) |
|  | Germany-Greifswald | | 106(33.7%) |  | Schizoaffective disorder | | 14(4.4%) |
|  | Netherlands | | 107(34.0%) |  | Other | | 35(11.1%) |
|  |  |  |  |  | Do not know | | 39(12.4%) |
| Mean DISCUS**^2^** | n=305 | Range: 0-2.73 | 0.73(0.63) | Stigma stress difference | n=310 | Range: -6-6 | -0.34(2.65) |
| PHQ total | n=308 | Range: 0-6 | 3.43(2.02) | Stigma conscious. total | n=306 | Range: 1-4 | 2.56(0.68) |
| PHQ categorical | Lower depression <3 | | 111(38.8%) | Mean Stopped seeing | n=307 | Range: 0-9 | 2.20(2.45) |
|  | Higher depression ≥3 | | 175(61.2%) | ISMI-10 total | n=312 | Range: 1-3.9 | 2.36(0.57) |
| BHS-4 total | n=307 | Range: 4-24 | 12.92(5.11) | ISMI categorical | Low internalized stigma 1.00-2.50 | | 190(60.3%) |
| BHS-4 categorical | Lower hopelessness ≤11 | | 102(32.4%) |  |  |  |  |
|  | Higher hopelessness >11 | | 205(65.1%) |  | High internalized stigma >2.50 | | 122(38.7%) |
| SIDAS total | n=305 | Range: 0-50 | 10.25(12.26) | WEMWBS total | n=312 | Range: 14-70 | 40.54(11.53) |
| SIDAS categorical | Not high risk SIDAS <21 | | 246(78.1%) | WEMWBS categorical | Lower mental wellbeing <41 | | 158(55.2%) |
|  | High risk SIDAS ≥21 | | 54(18.7%) |  | Higher mental wellbeing ≥42 | | 128(44.8%) |

| **Variable** | **N(%) or Mean(SD)** | | | **Variable** | **N(%) or Mean(SD)** | | |
| --- | --- | --- | --- | --- | --- | --- | --- |
| **Region = Eastern Europe (n=228)** | | | | | | | |
| Sex | Male | | 120(52.6%) | Told anyone diagnosis | Yes | | 210(92.5%) |
|  | Female | | 108(47.4%) |  | Yes, something else | | 13(5.7%) |
| Age | | | 43.51(11.68) |  | No | | 4(1.8%) |
| Years of education | | | 12.54(3.4) | Hospital admission for psychiatric care | Yes | | 182(79.8%) |
| Employment status | Full-time paid work | | 47(20.6%) |  | No | | 46(20.2%) |
|  | Part-time paid work | | 21(9.2%) | Age at first treatment | | | 24.35(11.14) |
|  | Self-employed | | 7(3.1%) | Know diagnosis | Yes | | 211(92.5%) |
|  | Unpaid‎/ voluntary | | 10(4.4%) |  | No | | 17(7.5%) |
|  | Unemployed | | 45(19.7%) | Diagnosis as reported by participants**^1^** | Schizophrenia | | 123(54.2%) |
|  | Retired | | 41(18%) |  | Depression | | 39(17.2%) |
|  | Homemaker | | 10(4.4%) |  | Anxiety disorder | | 27(11.9%) |
|  | Student | | 14(6.1%) |  | Bipolar disorder | | 19(8.4%) |
|  | Illness‎/sick leave | | 6(2.6%) |  | Psychosis | | 14(6.2%) |
|  | Other | | 27(11.8%) |  | PTSD | | 0(0%) |
| Country | Turkey - Ankara | | 105(46.1%) |  | Personality disorder | | 2(0.9%) |
|  | Czech republic | | 42(18.4%) |  | Schizoaffective disorder | | 5(2.2%) |
|  | Hungary | | 30(13.2%) |  | Other | | 1(0.4%) |
|  | Turkey - Istanbul | | 51(22.4%) |  | Do not know | | 17(7.5%) |
| Mean DISCUS**^2^** | n=215 | Range: 0-2.36 | 0.62(0.55) | Stigma stress difference | n=226 | Range: -6-6 | -1.04(3.13) |
| PHQ total | n=227 | Range: 0-6 | 2.33(1.79) | Stigma conscious. total | n=225 | Range: 1-4 | 2.44(0.74) |
| PHQ categorical | Lower depression <3 | | 132 (58.1%) | Stopped self total | n=228 | Range: 0-9 | 2.2(2.23) |
|  | Higher depression ≥3 | | 95(41.9%) | ISMI-10 total | n=215 | Range: 1-3.6 | 2.25(0.60) |
| BHS-4 total | N=222 | Range: 4-24 | 10.8(5.03) | ISMI categorical | Low internalized stigma 1.00-2.50 | | 151(70.2%) |
| BHS-4 categorical | Lower hopelessness ≤11 | | 111(50%) |  |  |  |  |
|  | Higher hopelessness >11 | | 111(50%) |  | High internalized stigma >2.50 | | 64(29.8%) |
| SIDAS total | n=225 | Range: 0-44 | 5.42(9.32) | WEMWBS total | n=223 | Range: 15-70 | 47.85(11.53) |
| SIDAS categorical | Not high risk SIDAS <21 | | 205(91.1%) | WEMWBS categorical | Lower mental wellbeing <41 | | 70(31.4%) |
|  | High risk SIDAS ≥21 | | 20(8.9%) |  | Higher mental wellbeing ≥42 | | 153(68.6%) |

| **Variable** | **N(%) or Mean(SD)** | | | **Variable** | **N(%) or Mean(SD)** | | |
| --- | --- | --- | --- | --- | --- | --- | --- |
| **Region = Southern Europe (n=271)** | | | | | | | |
| Sex | Male | | 154(56.8%) | Told anyone diagnosis | Yes | | 225(83.3%) |
|  | Female | | 117(43.2%) |  | Yes, something else | | 11(4.1%) |
| Age | | | 47.92(11.84) |  | No | | 34(12.6) |
| Years of education | | | 10.09(4.17) | Hospital admission for psychiatric care | Yes | | 212(78.2%) |
| Employment status | Full-time paid work | | 38(14%) |  | No | | 59(21.8%) |
|  | Part-time paid work | | 21(7.7%) | Age at first treatment | | | 28.71(11.07) |
|  | Self-employed | | 5(1.8%) | Know diagnosis | Yes | | 209(77.4%) |
|  | Unpaid‎/ voluntary | | 1(0.4%) |  | No | | 61(22.6%) |
|  | Unemployed | | 71(26.2%) | Diagnosis as reported by participants**^1^** | Schizophrenia | | 52(19.5%) |
|  | Retired | | 42(15.5%) |  | Depression | | 53(19.9%) |
|  | Homemaker | | 17(6.3%) |  | Anxiety disorder | | 15(5.6%) |
|  | Student | | 9(3.3%) |  | Bipolar disorder | | 63(23.6%) |
|  | Illness‎/sick leave | | 39(14.4%) |  | Psychosis | | 16(6.0%) |
|  | Other | | 28(10.3%) |  | PTSD | | 0(0%) |
| Country | Catalonia | | 43(15.9%) |  | Personality disorder | | 6(2.2%) |
|  | Italy-Brescia | | 46(17%) |  |  |  |  |
|  | Italy-Naples | | 50(18.5%) |  | Schizoaffective disorder | | 14(5.2%) |
|  | Italy-Verona | | 53(19.6%) |  |  |  |  |
|  | Spain-Madrid | | 49(18.1%) |  | Other | | 4(1.5%) |
|  | Portugal | | 30(11.1%) |  | Do not know | | 60(22.5%) |
| Mean DISCUS**^2^** | n=267 | Range: 0-2 | 0.38(0.41) | Stigma stress difference | n=270 | Range: -6-6 | -0.66(3.23) |
| PHQ total | n=271 | Range: 0-6 | 2.38(1.92) | Stigma conscious. total | n=270 | Range: 1-4 | 2.46(0.68) |
| PHQ categorical | Lower depression <3 | | 170(62.7%) | Stopped self total | n=271 | Range:0-9 | 1.21(1.7) |
|  | Higher depression ≥3 | | 101(37.3%) | ISMI-10 total | n=267 | Range: 1.10-4 | 2.2(0.55) |
| BHS-4 total | n=269 | Range: 4-24 | 11.31(5.42) | ISMI categorical | Low internalized stigma 1.00-2.50 | | 205(76.8) |
| BHS-4 categorical | Lower hopelessness ≤11 | | 128(47.4%) |  |  |  |  |
|  | Higher hopelessness >11 | | 142(52.6%) |  | High internalized stigma >2.50 | | 62(23.2%) |
| SIDAS total | n=245 | Range: 0-48 | 8.11(10.01) | WEMWBS total | n=270 | Range:18-70 | 44.74(11.40) |
| SIDAS categorical | Not high risk suicide behaviour <21 | | 224(89.6%) | WEMWBS categorical | Lower mental wellbeing <41 | | 96(35.6%) |
|  | High risk suicide behaviour ≥21 | | 26(10.4%) |  | Higher mental wellbeing ≥42 | | 174(64.4%) |

| **Variable** | **N(%) or Mean(SD)** | | | **Variable** | **N(%) or Mean(SD)** | | |
| --- | --- | --- | --- | --- | --- | --- | --- |
| **Region = Latin America(n=131)** | | | | | | | |
| Sex | Male | | 27(20.6%) | Told anyone diagnosis | Yes | | 108(82.4%) |
|  | Female | | 104(79.4%) |  | Yes, something else | | 10(7.6%) |
| Age | | | 45.44(11.13) |  | No | | 13(9.9%) |
| Years of education | | | 12.71(3.59) | Hospital admission for psychiatric care | Yes | | 30(22.9) |
| Employment status | Full-time paid work | | 42(32.1%) |  | No | | 101(77.1%) |
|  | Part-time paid work | | 17(13%) | Age at first treatment | | | 33.04(11.58) |
|  | Self-employed | | 6(4.6%) | Know diagnosis | Yes | | 119(90.8%) |
|  | Unpaid‎/ voluntary | | 1(0.8%) |  | No | | 12(9.2%) |
|  | Unemployed | | 14(10.7%) | Diagnosis as reported by participants**^1^** | Schizophrenia | | 6(4.6%) |
|  | Retired | | 14(10.7%) |  | Depression | | 74(56.5%) |
|  | Homemaker | | 2(1.5%) |  | Anxiety disorder | | 64(48.9%) |
|  | Student | | 4(3.1%) |  | Bipolar disorder | | 12(9.2%) |
|  | Illness‎/sick leave | | 29(22.1%) |  | Psychosis | | 0(0%) |
|  | Other | | 2(1.5%) |  | PTSD | | 0(0%) |
| Country | Brazil-Montes Claros | | 31(23.7%) |  | Personality disorder | | 0(0%) |
|  | Brazil-Sao Paulo | | 100(76.3%) |  | Schizoaffective disorder | | 0(0%) |
|  |  |  |  |  | Other | | 3(2.3%) |
|  |  |  |  |  | Do not know | | 12(9.2%) |
| Mean DISCUS**^2^** | n=131 | Range: 0-2.82 | 0.88(0.69) | Stigma stress difference | n=131 | Range: -6-6 | 0.80(3.39) |
| PHQ total | n=131 | Range: 0-6 | 4.18(1.93) | Stigma conscious. total | n=131 | Range: 1-4 | 2.06(0.78) |
| PHQ categorical | Lower depression <3 | | 33(25.2%) | Stopped self total | n=111 | Range: 0-9 | 2.66(2.88) |
|  | Higher depression ≥3 | | 98(74.8%) | ISMI-10 total | n=130 | Range: 1-3.3 | 2.23(0.49) |
| BHS-4 total | n=131 | Range: 4-24 | 11.82(5.25) | ISMI categorical | Low internalized stigma 1.00-2.50 | | 93(71.5) |
| BHS-4 categorical | Lower hopelessness ≤11 | | 54(41.2%) |  |  |  |  |
|  | Higher hopelessness >11 | | 77(58.8%) |  | High internalized stigma >2.50 | | 37(28.5%) |
| SIDAS total | n=131 | Range: 0-48 | 12.08(14.35) | WEMWBS total | n=130 | Range: 16-70 | 40.73(12.92) |
| SIDAS categorical | Not high risk suicide behaviour <21 | | 101(77.1%) | WEMWBS categorical | Lower mental wellbeing <41 | | 74(56.9%) |
|  | High risk suicide behaviour ≥21 | | 30(22.9%) |  | Higher mental wellbeing ≥42 | | 56(43.1%) |

**^1^**Note as an inclusion criterion all patients had a clinician-reported primary diagnosis of either schizophrenia, depression, bipolar disorder or anxiety disorder. The categories provided here represent the diagnoses reported by participants

**^2^**Median and inter-quartile range presented rather than mean(SD) due to non-normality of scores

**Supplementary Table 2**: Frequency and percentage response category endorsement for each DISCUS item by region (n=1,169)

| **DISCUS Item** | **Region** | **N** | **Non applicable (%)** | **Not at all (%)** | **A little (%)** | **Moderately (%)** | **A lot (%)** |
| --- | --- | --- | --- | --- | --- | --- | --- |
| DISCUS 1 - Making or keeping friends | Total sample | 1195 | 6.0 | 46.2 | 17.6 | 16.9 | 12.7 |
|  | Africa | 132 | 3.8 | 56.8 | 22.0 | 6.1 | 11.4 |
|  | Asia | 118 | 5.9 | 44.9 | 20.3 | 16.1 | 12.7 |
|  | Western Europe | 315 | 10.2 | 35.9 | 14.0 | 22.9 | 14.9 |
|  | Eastern Europe | 228 | 3.1 | 49.6 | 18.0 | 17.5 | 11.8 |
|  | Southern Europe | 271 | 5.9 | 54.2 | 21.0 | 9.6 | 9.2 |
|  | Latin America | 131 | 3.8 | 38.9 | 11.5 | 28.2 | 17.6 |
| DISCUS 2 - Dating or intimate relationships | Total sample | 1195 | 17.7 | 44.8 | 12.5 | 10.7 | 12.6 |
|  | Africa | 132 | 15.9 | 54.5 | 12.1 | 8.3 | 8.3 |
|  | Asia | 118 | 40.7 | 44.9 | 3.4 | 4.2 | 6.8 |
|  | Western Europe | 315 | 19.7 | 31.1 | 14.6 | 14.6 | 17.1 |
|  | Eastern Europe | 228 | 9.6 | 45.6 | 14.9 | 11.0 | 14.5 |
|  | Southern Europe | 271 | 18.8 | 53.5 | 12.9 | 8.5 | 6.3 |
|  | Latin America | 131 | 6.1 | 48.1 | 10.7 | 13.7 | 21.4 |
| DISCUS 3 - Housing | Total sample | 1195 | 27.9 | 56.2 | 5.9 | 5.0 | 4.0 |
|  | Africa | 132 | 22.0 | 61.4 | 9.1 | 4.5 | 3.0 |
|  | Asia | 118 | 31.4 | 52.5 | 0.8 | 6.8 | 8.5 |
|  | Western Europe | 315 | 29.2 | 50.2 | 6.7 | 5.7 | 5.1 |
|  | Eastern Europe | 228 | 4.8 | 82.5 | 5.7 | 3.5 | 3.5 |
|  | Southern Europe | 271 | 55.4 | 36.2 | 4.8 | 2.2 | 1.1 |
|  | Latin America | 131 | 11.5 | 64.9 | 7.6 | 10.7 | 5.3 |
| DISCUS 4 - Education | Total sample | 1195 | 29.3 | 46.8 | 8.8 | 6.3 | 6.9 |
|  | Africa | 132 | 18.2 | 59.1 | 11.4 | 4.5 | 2.3 |
|  | Asia | 118 | 63.6 | 22.0 | 2.5 | 6.8 | 3.4 |
|  | Western Europe | 315 | 28.6 | 38.1 | 10.2 | 10.5 | 9.5 |
|  | Eastern Europe | 228 | 5.7 | 65.4 | 11.8 | 4.8 | 10.5 |
|  | Southern Europe | 271 | 46.9 | 39.5 | 8.1 | 2.2 | 2.6 |
|  | Latin America | 131 | 16.0 | 60.3 | 4.6 | 8.4 | 10.7 |
| DISCUS 5 - Finding a job | Total sample | 1195 | 27.8 | 44.7 | 9.6 | 8.5 | 8.6 |
|  | Africa | 132 | 21.2 | 54.5 | 15.2 | 3.8 | 5.3 |
|  | Asia | 118 | 44.9 | 26.3 | 4.2 | 15.3 | 9.3 |
|  | Western Europe | 315 | 29.8 | 37.1 | 8.9 | 11.1 | 10.2 |
|  | Eastern Europe | 228 | 12.3 | 51.3 | 14.0 | 8.8 | 13.6 |
|  | Southern Europe | 271 | 39.5 | 44.6 | 8.5 | 4.4 | 2.6 |
|  | Latin America | 131 | 16.8 | 58.0 | 5.3 | 8.4 | 11.5 |
| DISCUS 6 - Keeping a job | Total sample | 1195 | 24.7 | 42.3 | 9.8 | 9.8 | 12.3 |
|  | Africa | 132 | 25.8 | 56.1 | 8.3 | 3.0% | 6.8% |
|  | Asia | 118 | 38.1 | 39.8 | 5.1 | 10.2% | 6.8% |
|  | Western Europe | 315 | 28.3 | 36.8 | 7.9 | 10.8% | 12.4% |
|  | Eastern Europe | 228 | 11.8 | 49.1 | 13.2 | 11.0% | 14.5% |
|  | Southern Europe | 271 | 33.6 | 41.0 | 11.1 | 5.5 | 8.5 |
|  | Latin America | 131 | 6.9 | 34.4 | 11.5 | 20.6 | 26.7 |
| DISCUS 7 - Social life | Total sample | 1195 | 6.7 | 56.1 | 14.6 | 11.4 | 10.2 |
|  | Africa | 132 | 4.5 | 57.6 | 14.4 | 6.8 | 13.6 |
|  | Asia | 118 | 4.2 | 68.6 | 3.4 | 8.5 | 14.4 |
|  | Western Europe | 315 | 12.4 | 43.2 | 13.7 | 16.2 | 12.1 |
|  | Eastern Europe | 228 | 2.2 | 65.4 | 18.0 | 7.9 | 6.6 |
|  | Southern Europe | 271 | 6.3 | 64.6 | 17.7 | 7.0 | 4.4 |
|  | Latin America | 131 | 6.1 | 40.5 | 14.5 | 22.1 | 16.8 |
| DISCUS 8 - Privacy | Total sample | 1195 | 6.4 | 68.6 | 10.1 | 7.2 | 6.7 |
|  | Africa | 132 | 3.0 | 75.0 | 8.3 | 4.5 | 8.3 |
|  | Asia | 118 | 0.0 | 90.7 | 6.8 | 0.8 | 1.7 |
|  | Western Europe | 315 | 18.4 | 53.0 | 8.6 | 9.8 | 7.6 |
|  | Eastern Europe | 228 | 2.2 | 75.4 | 9.2 | 7.9 | 5.3 |
|  | Southern Europe | 269 | 3.0 | 74.9 | 14.4 | 2.6 | 4.4 |
|  | Latin America | 131 | 1.5 | 55.0 | 11.5 | 17.6 | 14.5 |
| DISCUS 9 - Personal safety and security | Total sample | 1195 | 4.9 | 63.6 | 10.0 | 10.6 | 10.1 |
|  | Africa | 132 | 1.5 | 72.0 | 8.3 | 12.1 | 6.1 |
|  | Asia | 118 | 0.8 | 64.4 | 14.4 | 11.0 | 9.3 |
|  | Western Europe | 315 | 12.1 | 47.6 | 8.9 | 13.7 | 15.2 |
|  | Eastern Europe | 228 | 2.6 | 70.2 | 13.6 | 7.0 | 6.6 |
|  | Southern Europe | 271 | 3.3 | 76.0 | 9.6 | 5.2 | 5.9 |
|  | Latin America | 131 | 2.3 | 55.7 | 5.3 | 19.1 | 17.6 |
| DISCUS 10 - Starting a family/  having children | Total sample | 1195 | 37.6 | 44.5 | 5.3 | 5.7 | 6.1 |
|  | Africa | 132 | 25.8 | 61.4 | 8.3 | 1.5 | 3.0 |
|  | Asia | 118 | 59.3 | 23.7 | 3.4 | 5.9 | 7.6 |
|  | Western Europe | 315 | 38.7 | 38.7 | 4.8 | 7.9 | 7.0 |
|  | Eastern Europe | 228 | 25.4 | 52.2 | 6.1 | 7.5 | 8.8 |
|  | Southern Europe | 271 | 52.4 | 36.5 | 5.9 | 1.5 | 3.3 |
|  | Latin America | 115 | 17.6 | 63.4 | 2.3 | 9.9 | 6.9 |
| DISCUS 11 - Avoided or shunned at work by people who know you have mental health problem | Total sample | 1195 | 3.8 | 46.9 | 19.2 | 17.2 | 12.3 |
|  | Africa | 132 | 3.0 | 51.5 | 17.4 | 14.4 | 13.6 |
|  | Asia | 118 | 2.5 | 53.4 | 16.9 | 15.3 | 11.9 |
|  | Western Europe | 315 | 9.8 | 35.2 | 18.4 | 19.0 | 14.9 |
|  | Eastern Europe | 228 | 1.3 | 41.7 | 25.9 | 20.6 | 10.5 |
|  | Southern Europe | 271 | 1.1 | 59.8 | 19.9 | 12.5 | 6.6 |
|  | Latin America | 131 | 0.8 | 46.6 | 11.5 | 21.4 | 19.8 |
